# Unraveling Botulinum Neurotoxin A Light-Chain-Induced Signaling Pathways: A Phosphoproteomic Analysis in a Controlled Cellular Model

**DOI:** 10.3390/ijms26115168

**Published:** 2025-05-28

**Authors:** Chensi Zhu, Liangyan Zhang, Wenjing Yu, Yeqing Tu, Xiaolan Yang, Deyu Li, Hui Wang, Tao Li

**Affiliations:** State Key Laboratory of Pathogen and Biosecurity, Academy of Military Medical Sciences, Beijing 100071, China; chace3033@outlook.com (C.Z.); polini@live.cn (L.Z.); cpu_ywj@163.com (W.Y.); tyq2962280944@163.com (Y.T.); yangxiaolanyxl@163.com (X.Y.); 18842345409@163.com (D.L.)

**Keywords:** botulinum neurotoxin, phosphorylation, microarray

## Abstract

Botulinum neurotoxin type A (BoNT/A), among the most potent known toxins, is widely used in cosmetic medicine. However, its toxicity mechanisms remain poorly understood due to a lack of suitable models. Here, we generated a doxycycline (DOX)-inducible Neuro-2a cell line stably expressing the BoNT/A light chain (ALC). ALC expression was confirmed by GFP and FLAG tag antibodies, and its activity was validated through cleavage of the substrate SNAP-25. Using this model, combined with natural toxin infection of cells, phospho-antibody microarray analysis revealed significant alterations in host phosphorylation networks in both ALC-expressing and toxin-infected cells. Among the shared phosphorylation changes, 75 proteins showed upregulation, while 27 were downregulated. Upregulated phosphorylation events were enriched in pathways such as PI3K-AKT signaling, EGFR tyrosine kinase inhibitor resistance, and Ras signaling, whereas downregulated events were associated with the ERBB and thyroid hormone signaling pathways. Key alterations were observed in AKT signaling, with protein–protein interaction analysis identifying Hsp90ab1 and Map2k1 as central hub molecules for upregulated and downregulated proteins, respectively. This study establishes a robust Neuro-2a-based model system to study BoNT/A toxicity and provides insights into toxin-induced phosphorylation network changes, offering a valuable platform for therapeutic screening and mechanistic exploration.

## 1. Introduction

Botulinum neurotoxin type A (BoNT/A) is the most potent biological toxin known [1], with a lethal inhalation dose of 0.7–0.9 µg in a 70 kg adult and an intravenous LD50 of 90–150 ng [2]. This extreme toxicity has led to its classification as a Category A bioterrorism agent by the U.S. Centers for Disease Control and Prevention [3]. BoNT/A is a bi-chain protein consisting of a 50 kDa light chain (LC) and a 100 kDa heavy chain (HC) [4]. It binds to presynaptic membrane receptors and enters neurons via receptor-mediated endocytosis. Following endosomal acidification, the HC’s N-terminus forms a translocation channel, releasing the LC into the cytosol [5]. The LC specifically cleaves SNAP-25, a SNARE protein essential for vesicle fusion, leading to neurotransmitter release inhibition and muscle paralysis [6]. Despite its clinical applications in treating dystonic disorders and cosmetic uses [7,8,9], current treatments for BoNT/A poisoning are limited. Equine antitoxin, though commonly used, has a narrow therapeutic window [10], and severe cases requiring mechanical ventilation are both costly and resource-intensive.

Traditional studies of BoNT/A have primarily relied on cell-free systems using purified toxin proteins [11,12,13]. While these systems have provided valuable insights into the basic biochemical properties of the toxin, they have limitations in simulating the dynamic behavior of the toxin inside living cells, particularly in studying its prolonged effects. To address these limitations, we have established a doxycycline-inducible Neuro-2a cell line [14] stably expressing the BoNT/A light chain (ALC). This cell model more closely mimics the physiological conditions of toxin action and provides a robust system for studying the intracellular mechanisms of ALC.

However, the interactions of ALC with host molecules beyond its well-characterized target, SNAP-25 [15], remain poorly understood. The host cellular response to intracellular ALC, as well as the mechanisms underlying its resistance to degradation within neurons, are yet to be elucidated. Combining this cell model with phosphorylation antibody microarray analysis, we aim to systematically investigate the changes in the host phosphorylation network induced by ALC. This approach not only provides a foundation for understanding the molecular mechanisms of ALC toxicity but also opens new avenues for the development of targeted therapeutic interventions.

## 2. Results

### 2.1. The Cell Line Model Successfully Mimics the Activity of the Botulinum Neurotoxin Type A Light Chain

**Figure 1:**
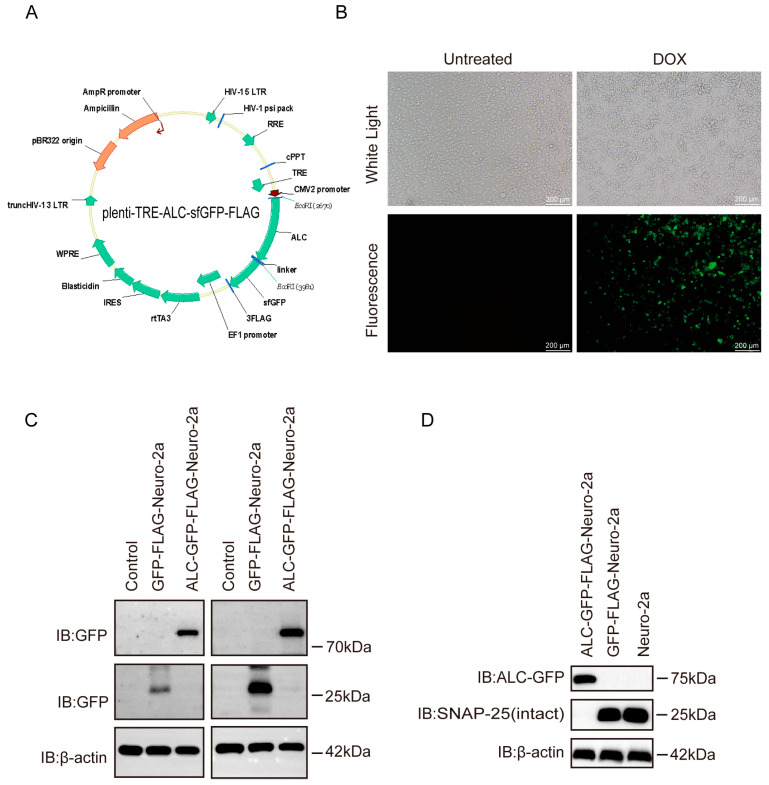
Characterization of the ALC-GFP-FLAG-Neuro-2a stable cell line. (**A**), plenti-TRE-ALC-sfGFP-FLAG plasmid map. IB, immunoblotting. (**B**) Fluorescence expression of the cell line observed under a microscope. (**C**) Detection of protein expression in the stable cell line using GFP and FLAG antibodies. (**D**) Demonstration of the stable cell line’s ability to cleave the substrate SNAP-25. β-actin served as a loading control to normalize protein input across lanes.

To establish a robust model for studying the intracellular activity of ALC, we developed a stable cell line with a doxycycline-inducible expression system (Figure 1A). This system was designed to tightly regulate ALC expression under the control of a Tet-On promoter [16], ensuring precise and reproducible induction of the toxin. The ALC protein was fused to GFP (green fluorescent protein) to enable real-time visualization of its expression and localization within living cells. Additionally, a FLAG tag was incorporated for immunodetection, allowing us to confirm protein expression through multiple experimental approaches.

Upon induction with doxycycline, the stable cell line exhibited distinct green fluorescence under fluorescence microscopy, confirming the successful expression of the ALC-GFP fusion protein (Figure 1B). This fluorescence serves as a direct and reliable visual marker for ALC expression, allowing us to monitor its localization and activity in a cellular context. Western blot analysis further validated the expression of ALC, as both GFP and FLAG antibodies effectively detected the protein (Figure 1C). This dual-detection approach ensures the specificity and authenticity of ALC expression in the cell line.

Notably, only the ALC-GFP-FLAG demonstrated substrate cleavage activity, whereas the GFP-FLAG showed no cleavage (Figure 1D). This result underscores the specificity of the stable cell line and confirms that the observed cleavage activity is directly attributable to ALC expression. The design of this cell line ensures that ALC is expressed only upon doxycycline induction, minimizing potential off-target effects and enabling precise control over toxin activity for downstream experiments.

In summary, we have successfully established a doxycycline-inducible ALC-GFP-FLAG-Neuro-2a cell line, which serves as a valuable tool for investigating the host phosphorylation network changes induced by ALC expression.

### 2.2. Phosphorylation Antibody Array Workflow Validation and Venn Diagram Analysis

**Figure 2:**
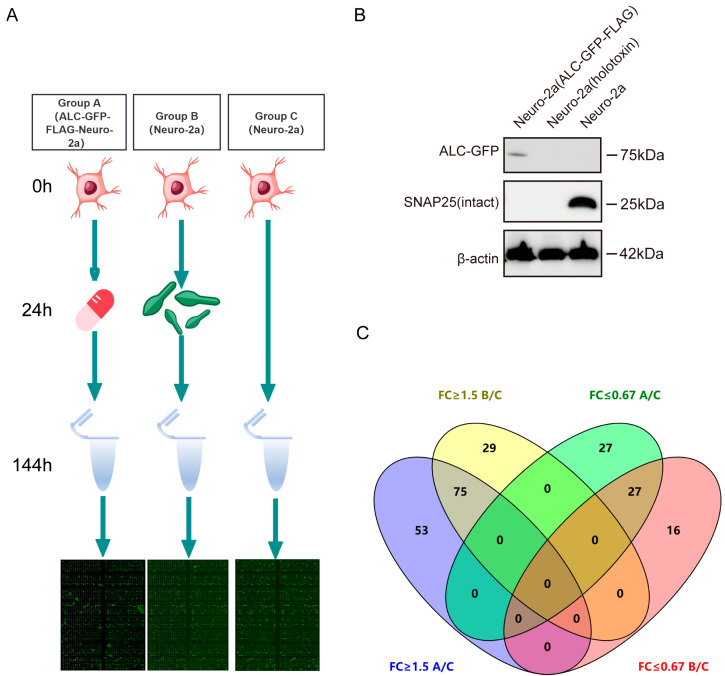
Experimental workflow and validation. (**A**) Flowchart of the phosphorylation antibody array experiment. At the 0 h time point, cells were seeded. At the 24 h time point, group A received doxycycline to induce gene expression, while group B received BoNT/A. At the 48 h time point, DOX was removed from group A. At the 144 h time point, samples were collected for antibody microarray analysis. (**B**) Validation of the stable cell line expressing the ALC and its ability to cleave the substrate, demonstrating functionality comparable to the full toxin. (**C**) Venn diagram analysis of proteins with phosphorylation changes (upregulated by ≥50% or downregulated by ≤67%) to identify proteins consistently upregulated or downregulated across both experimental groups. FC: fold change.

- To investigate the host responses and associated signaling pathways triggered by the intracellular entry of ALC, we conducted a phosphoproteomics analysis using a commercial antibody microarray (Figure 2A). This array is widely applied to identify key signaling proteins that undergo phosphorylation changes in biological processes [17,18,19,20]. The array covers 30 distinct signaling pathways, enabling the detection of 584 phosphorylation sites across 332 key signaling proteins. Our experimental design comprises three groups: Group A, Group B, and Group C.
- Group A consisted of ALC-GFP-FLAG-Neuro-2a cells where ALC was stably expressed as a GFP-FLAG fusion protein under doxycycline-inducible control. Doxycycline (10 µg/mL) was administered at the 24 h time point to induce ALC expression, allowing us to study the effects of ALC in a tightly regulated and inducible manner. This setup provided a controlled system to examine the intracellular effects of ALC expression independent of the toxin delivery complex. Group B utilized wild-type Neuro-2a cells treated with BoNT/A at a concentration of 1 ng/mL. This group simulated the natural process of ALC entering host cells through BoNT/A-mediated delivery, reflecting the physiological route of ALC intracellular activity. Group C served as the control group, utilizing untreated wild-type Neuro-2a cells to establish a baseline for phosphorylation levels in the absence of ALC.
- The key focus of this experimental design was to compare the phosphorylation profiles between Group B (natural ALC delivery via BoNT/A) and Group A (controlled ALC expression), with Group C as a reference. By identifying the common phosphorylation changes in both experimental groups, we aimed to elucidate the conserved host signaling networks that are specifically activated by ALC’s intracellular activity, regardless of the delivery method. Such proteins are postulated to influence the prolonged intracellular stability of the Botulinum neurotoxin type A light chain [21]. Western blot analysis confirmed the successful expression of ALC in the stable cell line and the intracellular entry of botulinum neurotoxin type A, as evidenced by substrate cleavage (Figure 2B).
- The level of protein phosphorylation in each group was expressed as the ratio of phosphorylated to non-phosphorylated proteins. A significant difference in the phosphorylation ratio between groups was considered to exist when the ratio in the experimental groups was ≥1.5 or ≤0.67 that of the control group. In Group A, 128 proteins showed elevated phosphorylation levels, while 54 proteins exhibited reduced phosphorylation levels, with the maximum ratio reaching 7.17. In Group B, 104 proteins demonstrated increased phosphorylation levels, and 43 proteins showed decreased levels, with the maximum ratio being 9.08 (Supplementary Table S1).
- To effectively visualize and analyze the overlap in phosphorylation changes between Group A and Group B, we employed a Venn diagram [22]. This graphical representation allowed us to clearly depict the intersection of upregulated and downregulated phosphorylation events between the two experimental groups. Venn diagram analysis revealed that 53 proteins showed elevated phosphorylation levels only in Group A, and 29 proteins showed elevated levels only in Group B. Additionally, 27 proteins exhibited reduced phosphorylation levels only in Group A, and 16 proteins showed reduced levels only in Group B. Furthermore, 75 proteins exhibited commonly upregulated phosphorylation levels, while 27 proteins showed commonly downregulated phosphorylation levels (Figure 2C). These core proteins with consistent phosphorylation changes in both groups are likely to represent key nodes in the signaling pathways directly modulated by ALC.

### 2.3. Enrichment Analysis of Proteins with Upregulated Phosphorylation Levels

**Figure 3:**
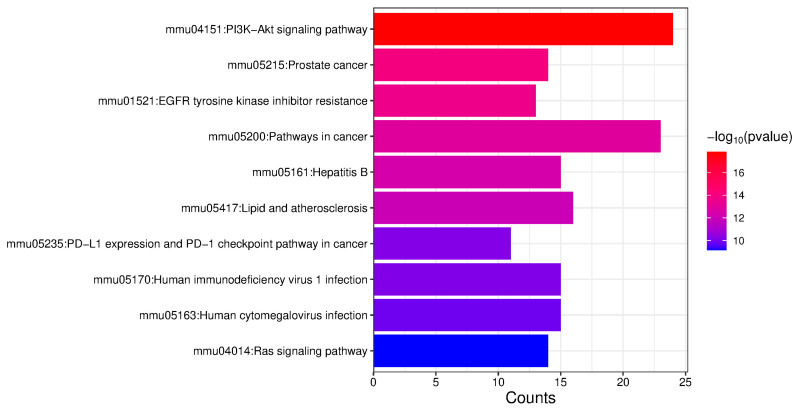
KEGG pathway enrichment analysis of proteins exhibiting upregulated phosphorylation levels. The top 10 enriched pathways are displayed.

The 75 proteins exhibiting upregulated phosphorylation levels, as identified through Venn diagram analysis, were subjected to Gene Ontology (GO) and KEGG pathway enrichment analysis using the DAVID database (https://davidbioinformatics.nih.gov/ (accessed on 22 March 2024)). The results revealed significant enrichment in the PI3K-AKT signaling pathway, EGFR tyrosine kinase inhibitor resistance, and Ras signaling pathway (Figure 3).

**Figure 4:**
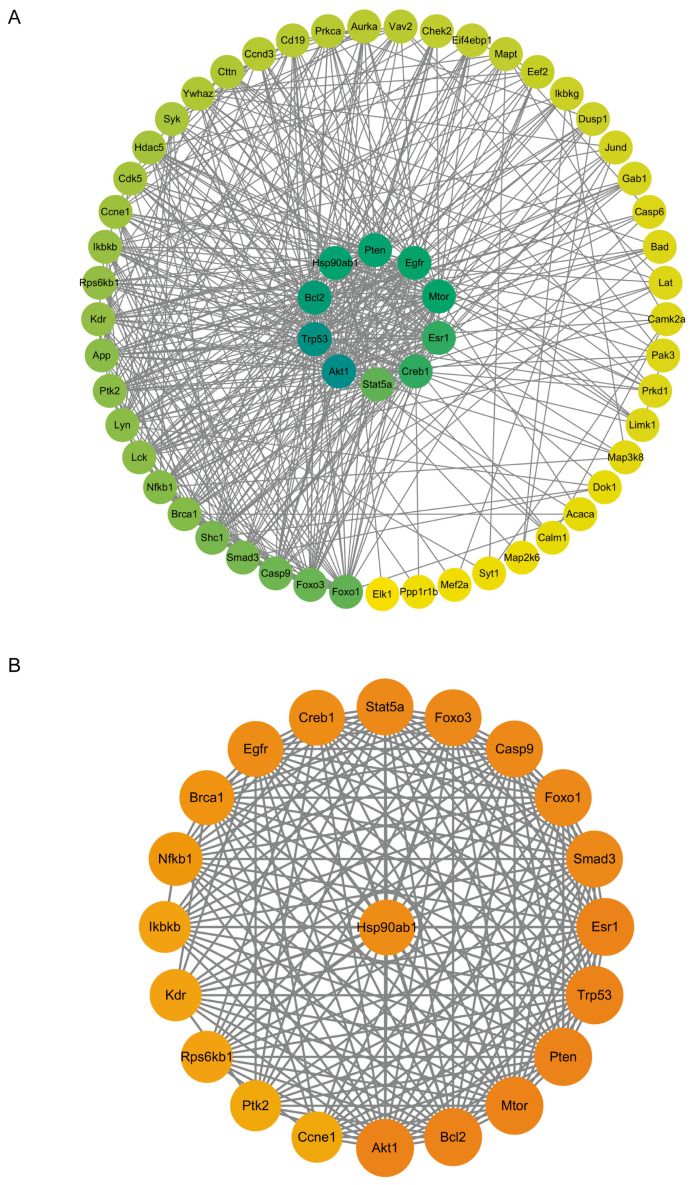
Protein–protein interaction (PPI) network analysis of proteins with upregulated phosphorylation levels. (**A**) PPI network of proteins exhibiting upregulated phosphorylation levels. (**B**) Subnetwork identified through MCODE plugin analysis of proteins with upregulated phosphorylation levels.

The proteins exhibiting upregulated phosphorylation levels were input into the STRING database (https://cn.string-db.org/ (accessed on 22 March 2024)) to construct a protein–protein interaction (PPI) network, which was subsequently refined and ranked using Cytoscape 3.10.1 (https://cytoscape.org/ (accessed on 22 March 2024)). The MCODE plugin was employed to identify critical subnetworks. The results revealed that the network comprises 59 nodes and 477 edges (Figure 4A), with a clustering coefficient of 0.641. Further analysis using the MCODE plugin identified 1 subnetwork, consisting of 21 nodes and 185 edges, with HSP90AB1 serving as the seed gene (Figure 4B). HSP90 not only assists in the activation of AKT [23] but also stabilizes the light chain of botulinum toxin to enhance its enzymatic activity [24].

### 2.4. The Phosphorylation Levels of Several Key Molecules in the PI3K-AKT Signaling Pathway Were Significantly Upregulated

**Figure 5:**
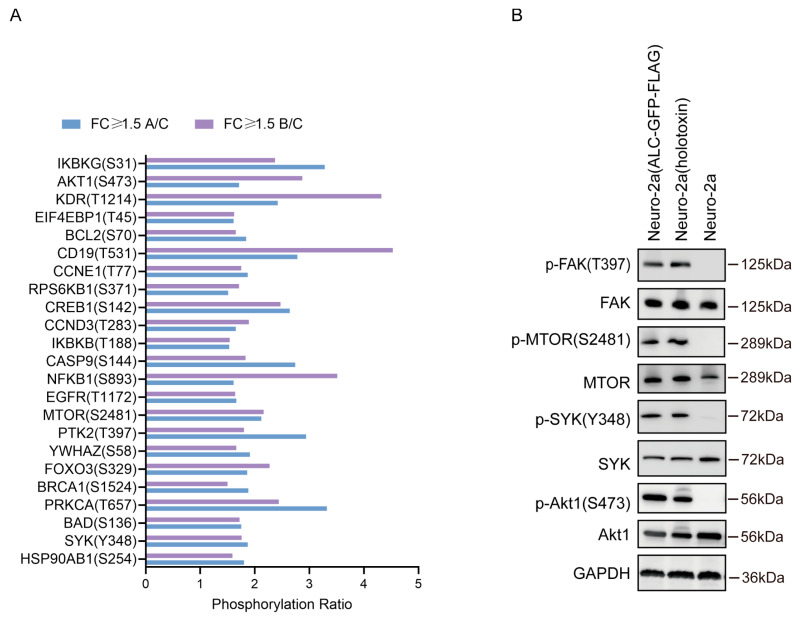
Analysis of phosphorylation changes in the Akt signaling pathway. (**A**) Relative changes in the phosphorylation levels of proteins enriched in the Akt signaling pathway in both experimental groups. (**B**) Representative Western blot images demonstrating the phosphorylation changes in FAK (T397), mTOR (S2481), SYK (Y348), and AKT1 (S473) in the two experimental groups.

Among the proteins enriched in the PI3K-AKT signaling pathway (Figure 5A), we further assessed the phosphorylation levels of several key molecules. Notably, the phosphorylation levels of FAK (T397), mTOR (S2481), AKT1 (S473), and SYK (Y348) were significantly upregulated in both experimental groups, while the protein expression levels remained unchanged, consistent with the antibody array results (Figure 5B). The PI3K-AKT and mTOR signaling pathways play critical roles in regulating cellular processes, including autophagy and apoptosis [25,26]. AKT inhibits apoptosis by phosphorylating and inactivating pro-apoptotic factors such as FOXO transcription factors, thereby promoting cell survival. Conversely, mTOR, particularly through its mTORC1 complex, suppresses autophagy by phosphorylating and inactivating key autophagic components. Under nutrient-rich conditions, mTORC1 is activated, inhibiting autophagy and promoting protein synthesis. However, under stress or nutrient deprivation, mTORC1 activity is suppressed, leading to autophagy induction. The interplay between AKT and mTOR signaling pathways tightly regulates cellular homeostasis, balancing survival and catabolic processes to maintain cell viability.

Focal adhesion kinase (FAK) is a non-receptor tyrosine kinase that plays a pivotal role in regulating cell survival, proliferation, and migration [27]. FAK has been shown to suppress autophagy through its interaction with integrin signaling and downstream pathways. Integrin-mediated activation of FAK promotes the phosphorylation of Src family kinases (SFKs), which subsequently activate the PI3K/AKT/mTOR axis [28,29].

SYK (spleen tyrosine kinase) plays a critical role in regulating cell survival by modulating apoptotic and autophagic pathways, with its effects varying depending on the cellular context and tumor type. In many cancers, SYK promotes cell survival by activating the PI3K/AKT signaling pathway, which stabilizes anti-apoptotic proteins such as MCL-1, BCL-XL, and XIAP. Specifically, SYK-mediated AKT activation inhibits GSK3 (glycogen synthase kinase 3), preventing the ubiquitination and proteasomal degradation of MCL-1, a key anti-apoptotic protein in chronic lymphocytic leukemia (CLL) and other malignancies. This mechanism has been demonstrated in B-cell lymphomas, where SYK inhibition leads to the destabilization of MCL-1 and subsequent apoptosis [30,31].

### 2.5. Enrichment Analysis of Proteins with Downregulated Phosphorylation Levels

**Figure 6:**
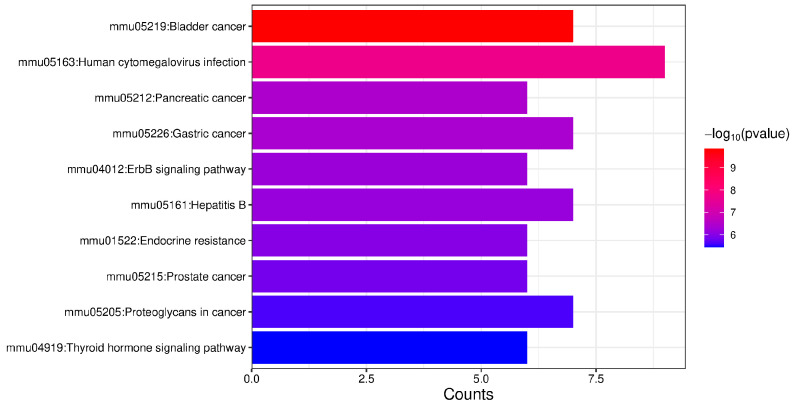
KEGG pathway enrichment analysis of proteins exhibiting downregulated phosphorylation levels. The top 10 enriched pathways are displayed.

For the 27 proteins exhibiting downregulated phosphorylation levels, pathway enrichment analysis revealed significant associations with bladder cancer, the ErbB signaling pathway, and the thyroid hormone signaling pathway (Figure 6).

**Figure 7:**
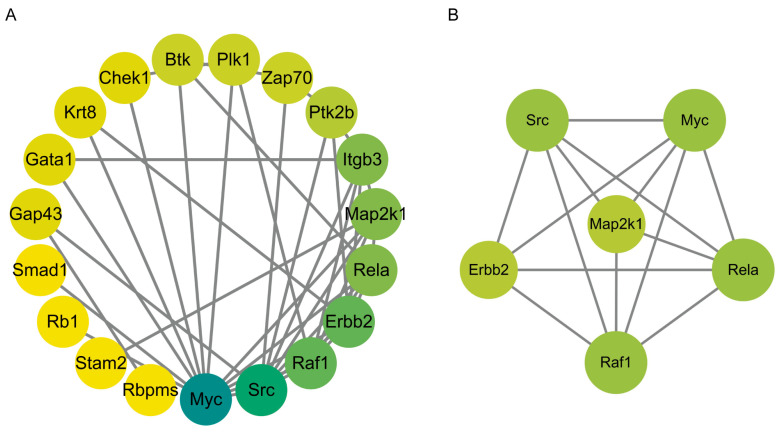
Protein–protein interaction (PPI) network analysis of proteins with downregulated phosphorylation levels. (**A**) PPI network of proteins exhibiting downregulated phosphorylation levels. (**B**) Subnetwork identified through MCODE plugin analysis of proteins with downregulated phosphorylation levels.

The proteins exhibiting downregulated phosphorylation levels were input into the STRING database to construct a protein–protein interaction (PPI) network (Figure 7A). The network comprises 19 nodes and 39 edges, with a clustering coefficient of 0.391. Further analysis using the MCODE plugin identified 1 subnetwork (Figure 7B), consisting of 21 nodes and 185 edges, with MAP2K1 serving as the seed gene.

## 3. Discussion

Botulinum neurotoxin type A (BoNT/A) is the most widely used toxin, with applications in cosmetic treatments, pain management, and the therapy of dystonic disorders [32,33,34]. However, it is also the most potent biological toxin, raising concerns about its potential use as a bioterrorism agent. Beyond its extreme toxicity, BoNT/A is characterized by an exceptionally long intracellular half-life, leading to prolonged muscle paralysis and significant harm [35]. The mechanisms by which BoNT/A evades intracellular protein degradation remain unclear, and effective interventions to counteract its effects in neurons are lacking. Current research on BoNT/A primarily focuses on its medical applications [36], while its mechanisms of action and host responses post-entry into the body are poorly understood. This study aims to elucidate the cellular responses triggered by the intracellular entry of the BoNT/A light chain protein, providing a foundation for further research. The Tet-On system, a small-molecule regulatory system responsive to tetracycline antibiotics, is widely used in molecular biology and genetic engineering for precise control of gene expression [37]. Given the trace lethality and detection challenges of BoNT/A, we constructed a doxycycline-regulated (tetracycline member family) Neuro-2a cell line stably expressing the BoNT/A light chain protein. This cell line allows controlled expression of the target gene, which can be easily detected via a tag protein. The induced light chain protein specifically cleaves SNAP-25, consistent with the enzymatic activity of native BoNT/A in neuronal cells, and serves as the basis for subsequent experiments. Compared to using the Flp-in/T-REX/293 system for expressing ALC [38], the lentiviral system offers significantly higher transfection efficiency and more stable gene expression [39]. Furthermore, constructing stable cell lines based on neuronal cells provides a more physiologically relevant model, accurately simulating the behavior of BoNT/A in neurons [40].

Protein phosphorylation is a central mechanism in cellular signal transduction, dynamically regulating protein activity, stability, and interactions to determine cell fate [41,42]. In pathogen–host interactions, pathogens often manipulate the host phosphorylation network to reshape the intracellular environment [43,44]. For example, *Mycobacterium tuberculosis* dephosphorylates phosphatidylinositol in the host cell membrane to inhibit cytokine release and pyroptosis in macrophages [45].

Src kinase plays a critical role in modulating the enzymatic activity and stability of ALC through tyrosine phosphorylation [46,47]. Studies demonstrate that Src phosphorylates tyrosine residues within the catalytic domains of BoNT serotypes A, B, and G, significantly enhancing their proteolytic activity and thermal stability [48]. Phosphorylation of ALC by Src stabilizes its structure, reduces autocatalytic degradation, and increases substrate affinity, while dephosphorylation reverses these effects. In neuronal cells, Src-mediated phosphorylation of internalized ALC correlates with prolonged toxin persistence, as shown in PC12 cells where Ca^2+^-dependent signaling enhances LC phosphorylation [11,49]. These findings suggest that Src-driven phosphorylation may be a key regulatory mechanism underlying the stability, intracellularity, and pathogenicity of BoNT. However, the precise mechanisms by which ALC influences the host cell’s phosphorylation network remain poorly understood and warrant systematic investigation.

Phosphorylation antibody arrays are high-throughput tools designed to detect protein phosphorylation states, covering key proteins across multiple signaling pathways [17]. They provide comprehensive information on global phosphorylation modifications, offering advantages such as high throughput, sensitivity, speed, and comprehensiveness. Using our stable cell line, we conducted high-throughput phosphorylation antibody array analysis. The results revealed that the intracellular entry of the ALC indeed induced changes in the host phosphorylation network, particularly in proteins within the PI3K-AKT signaling pathway. Specifically, the phosphorylation levels of FAK (T397), mTOR (S2481), AKT1 (S473), and SYK (Y348) were significantly upregulated in both experimental groups (Figure 4B). The PI3K-Akt signaling pathway is closely associated with autophagy and apoptosis [50,51,52,53]. According to the literature, phosphorylated Akt inhibits TSC1 and TSC2, leading to upregulated phosphorylation of mTORC1, which subsequently activates autophagy [25]. Additionally, activated Akt phosphorylates Bad and activates anti-apoptotic proteins such as Bcl-2 and Bax through multiple pathways [54], thereby inhibiting apoptosis.

The constructed protein–protein interaction network identified HSP90AB1 as the seed gene. HSP90 not only aids Akt activation [23] but is also crucial for the proper folding and enzymatic activity of the toxin [24]. It has been reported that geldanamycin, a specific inhibitor of HSP90, completely inhibits BoNT/A-mediated cleavage of SNAP-25 [55]. Based on these findings, we hypothesize that the BoNT/A light chain protein, upon entering the cell, upregulates the phosphorylation levels of HSP90AB1 and AKT by a specific mechanism that has not yet been elucidated. HSP90AB1 activation may stabilize both the toxin and AKT, potentially facilitating their functional maintenance. These interactions could suggest a coordinated mechanism for promoting cellular survival, as phosphorylated AKT activation appears to contribute to downstream signaling cascades associated with attenuation of autophagy and apoptosis pathways. Further studies are required to delineate whether this represents direct interaction or indirect regulatory effects. This may provide a new perspective on the exceptionally long intracellular half-life of the ALC, and the kinases in these signaling pathways could serve as potential targets for designing botulinum toxin inhibitors.

In summary, this study established a controllable, visualizable, and easily detectable stable cell line model expressing the BoNT/A light chain, which mimics the activity of the native toxin. Phosphorylation antibody array analysis revealed host responses triggered by the intracellular entry of the BoNT/A light chain, with enriched PI3K-AKT signaling pathways. These findings lay the groundwork for further research into the intracellular escape mechanisms of BoNT/A and the identification of potential therapeutic targets.

## 4. Materials and Methods

### 4.1. Generation of Stable Cell Lines

Neuro-2a (mouse neuroblastoma cell line) and 293T cell (human embryonic kidney cell line) cryovials were thawed in a 37 °C water bath for 1 min. Following thawing, the vials were centrifuged at 1200 rpm for 2 min at room temperature. The supernatant was discarded, and the cells were resuspended in DMEM complete medium (containing 10% fetal bovine serum) before being transferred to cell culture flasks. The flasks were then incubated at 37 °C in a 5% CO_2_ cell incubator. The ALC gene (residues 1–448 of BoNT/A, Uniport ID: Q7B8V4) [56] was PCR-amplified and subsequently cloned into the pLenti-TRE-ALC-GFP-FLAG lentiviral vector via EcoRI and BamHI restriction enzyme digestion with a flexible linker (GGGSGGGGS) to minimize steric hindrance while preserving C-terminal interactions critical for stability [57,58].

293T cells were seeded in 10 cm dishes. Once the cells reached 80% confluence, they were co-transfected with 16 µg of the lentiviral vector, 12 µg of the packaging plasmid psPAX2, and 4 µg of the envelope plasmid pMD2.G. The medium was changed 20 h post-transfection, and doxycycline (10 µg/mL) was added 24 h after the media change. Fluorescence was observed under a microscope 24 h later, and the cell supernatant containing the lentiviral particles was collected and stored at −80 °C.

Neuro-2a cells (2 × 10^5^ cells/well) were seeded in 6-well plates. When the cells reached 60% confluence, the frozen viral supernatant was added at an MOI of 100. The plates were incubated at 37 °C in a 5% CO_2_ incubator. The medium was changed 24 h post-infection, and doxycycline (10 µg/mL) was added 72 h post-infection. Fluorescence was observed under a microscope 24 h later. Following observation of fluorescence, the cells were trypsinized and expanded.

To obtain a clonal cell line, the infected Neuro-2a cells were seeded at a density of 1 × 10^4^ cells/well in 96-well plates. When the cells reached 60% confluence, blasticidin (2 µg/mL) was added for 7 days to select for stably transfected cells. The resulting clonal cell lines were then expanded and cryopreserved.

### 4.2. Detection of ALC-GFP-FLAG Protein Expression and Activity

Original Neuro-2a cells, Neuro-2a cells expressing GFP-FLAG, and Neuro-2a cells expressing ALC-GFP-FLAG were seeded at 5 × 10^5^ cells per well in 24-well plates. After 24 h of incubation, 10 µg/mL doxycycline was added to induce expression for 24 h. Cells were lysed on ice for 10 min using targetmoi RIPA Lysis Buffer (Strong) (containing 50 mM Tris pH 7.4, 150 mM NaCl, 1% Triton X-100, 1% sodium deoxycholate, 0.1% SDS), supplemented with targetmoi Protease Inhibitor Cocktail (EDTA-Free, 100× in DMSO) at a 1× working concentration. Proteins were separated by SDS-PAGE using 10% polyacrylamide gels under reducing conditions (constant voltage: 180 V, 50 min) and transferred to PVDF membranes via wet transfer at a constant current of 300 mA for 25 min. Proteins were separated by SDS-PAGE and transferred to PVDF membranes. Membranes were blocked with 5% (*w*/*v*) BD DIFCO™ Skim Milk in TBST (20 mM Tris-HCl, 150 mM NaCl, 0.05% Tween-20) for 1 h at room temperature. After blocking, membranes were incubated overnight at 4 °C with primary antibodies [SNAP-25 Rabbit mAb (Zen-Bioscience, Chengdu, China), Anti-GFP mouse mAb, Anti-DYKDDDDK mouse mAb, and Anti-β-actin mouse mAb (TransGen Biotech, Beijing, China)] diluted 1:1000 in blocking buffer. Following three TBST washes (10 min each), membranes were incubated for 1 h at room temperature with Goat Anti-Mouse IgG secondary antibody (TransGen Biotech) diluted 1:2000. After three additional TBST washes, protein bands were visualized using Super ECL Detection Reagent (Yeasen Biotech, Shanghai, China) and imaged on a Minichemi830 system (SINSAGE, Beijing, China) with 3 s of exposure time.

### 4.3. Phosphoproteomic Array

The experiment consisted of three groups: group A and group B served as experimental groups, utilizing ALC-GFP-FLAG-Neuro-2a cells and wild-type Neuro-2a cells, respectively, while group C served as a control group using wild-type Neuro-2a cells. At the 0 h time point, 5 × 10^6^ cells were seeded in 10 cm culture dishes. At the 24 h time point, group A received doxycycline (10 µg/mL) to induce gene expression, while group B received BoNT/A (1 ng/mL). The full-length BoNT/A was produced in our laboratory from a *Clostridium botulinum* culture, following the steps that have been described previously [59,60].

At the 48 h time point, DOX was removed from group A, followed by two washes with phosphate-buffered saline (PBS). At the 144 h time point, cells were lysed with Targetmoi RIPA Lysis Buffer (Strong) (50 mM Tris pH 7.4, 150 mM NaCl, 1% Triton X-100, 1% sodium deoxycholate, 0.1% SDS) supplemented with Targetmoi Protease Inhibitor Cocktail (EDTA-Free, 100× in DMSO; 1× working concentration) and Beyotime Phosphatase Inhibitor Cocktail (1× final concentration). Subsequent phospho-antibody microarray analysis (Catalog#PEX100) was conducted according to the Full Moon Biosciences standard operating procedure (https://www.fullmoonbio.com/product/phospho-explorer-antibody-array/ (accessed on 15 September 2021); info@fullmoonbio.com). Briefly, cell lysates were biotinylated and incubated with the antibody microarray at room temperature for 2 h. The microarray was then immersed in Cy3-streptavidin solution for 20 min, washed with deionized water, and scanned using SureScan Dx Microarray Scanner (Agilent Technologies Inc., Santa Clara, CA, USA). The level of protein phosphorylation in each group was expressed as the ratio of phosphorylated to non-phosphorylated proteins. A significant difference in the phosphorylation ratio between groups was considered to exist when the ratio in the experimental groups was ≥1.5 or ≤0.67 that of the control group.

### 4.4. Data Analysis of Phosphoproteomic Array

To identify proteins with significant differences in phosphorylation ratios between experimental groups, we input these proteins into Venny 2.1.0 (https://bioinfogp.cnb.csic.es/tools/venny/index.html (accessed on 22 March 2024)) to generate a Venn diagram [61,62]. This analysis seeks to pinpoint proteins whose phosphorylation levels are consistently upregulated or downregulated in both experimental settings. Subsequently, the results derived from the Venn diagram were input into the David database (https://david.ncifcrf.gov/ (accessed on 22 March 2024)) for KEGG pathway enrichment analysis [63]. The top 10 ranked entries were selected for presentation and further analysis. Additionally, the Venn diagram results were utilized to construct a protein–protein interaction network within the STRING database (https://cn.string-db.org/ (accessed on 22 March 2024)) [64]. This network was then imported into Cytoscape 3.10.1 ((https://cytoscape.org/ (accessed on 22 March 2024)) [65], where the Mcode plugin was employed to identify key genes [66].

## Data Availability

All data presented are available in the manuscript.

## Supplementary Materials

The following supporting information can be downloaded at: https://www.mdpi.com/article/10.3390/ijms26115168/s1.

## Abbreviations

The following abbreviations are used in this manuscript:

BoNT/A	Botulinum neurotoxin type A
DOX	Doxycycline
ALC	BoNT/A light chain
LC	Light chain
HC	Heavy chain
SNAP-25	Synaptosome-associated protein 25
PPI	Protein–protein interaction
TBST	Tris-buffered saline with Tween 20
GO	Gene Ontology
KEGG	Kyoto Encyclopedia of Genes and Genomes
IB	Immunoblotting
